# Vesiculobullous Diseases

**DOI:** 10.3390/medicina58020186

**Published:** 2022-01-26

**Authors:** Simo Huang, Sylvia Hsu, Kiran Motaparthi

**Affiliations:** 1 Department of Dermatology, Lewis Katz School of Medicine at Temple University, Philadelphia, PA 19140, USA; simo.huang@tuhs.temple.edu (S.H.); sylvia.hsu@tuhs.temple.edu (S.H.); 2 Department of Dermatology, University of Florida College of Medicine, Gainesville, FL 32606, USA

A diverse range of inflammatory dermatoses are characterized by vesicles or bullae. Vesiculobullous diseases can be associated with significant morbidity and mortality rates that necessitate their early clinical recognition and prompt management. While the diagnosis and management of vesiculobullous dermatoses may appear daunting to clinicians, we present an updated review regarding the scope of vesiculobullous disorders in detail, including more recent developments in diagnostic testing and targeted therapy.

This Special Issue highlights several vesiculobullous dermatoses that may be encountered in both clinic and hospital settings. One important focus is Stevens–Johnson syndrome/toxic epidermal necrolysis (SJS/TEN), a severe cutaneous adverse reaction (SCAR) and true dermatological emergency that can have profound associated morbidity and mortality rates [1]. In addition, erythema multiforme and fixed drug eruption, which share similar histopathologic features with SJS/TEN, are reviewed separately due to their significant differences in pathogenesis, clinical course, and management [2]. The diagnosis and management of the pemphigoid and pemphigus family of disorders are also included in this Special Issue. Other disease entities reviewed include dermatitis herpetiformis, linear IgA bullous dermatosis (LABD), acute generalized exanthematous pustulosis (AGEP), and pustular psoriasis, which can all be categorized under vesiculobullous dermatoses.

A comprehensive understanding of the clinical presentation and pathogenesis of these diseases is key to diagnosis. Morphology and distribution are important aspects of the visual examination highlighted in this Special Issue. While every dermatosis discussed herein can present with vesicles or bullae, they may be described in several ways, and their varied clinical presentation is a direct result of their unique pathogenesis. The dusky necrosis seen in erythema multiforme, fixed drug eruption, and SJS/TEN results from an interface reaction pattern causing epidermal necrosis [3,4,5]. The flaccid bullae in pemphigus dermatoses contrast from the tense bullae in pemphigoid dermatoses due to differing antigenic targets in the epidermis and dermoepidermal junction [6,7]. Non-follicular sterile pustules develop from neutrophilic infiltration of the epidermis and should invoke pustular psoriasis or AGEP [8]. The distribution of lesions is also a vital clue for clinicians when morphology may be equivocal. Dusky, targetoid lesions may be seen in SJS/TEN, erythema multiforme, and fixed drug eruption, but a predilection for symmetric involvement of the head and acral surfaces should favor erythema multiforme [3]. In another example, lesions with a predominantly seborrheic involvement should prompt the clinician to consider pemphigus foliaceus when the morphology is ambiguous [9]. Noting the morphology and distribution of lesions helps to narrow down differential diagnoses before verbal communication between the patient and clinician even starts. These aspects of the clinical presentation and pathogenesis are explored in-depth in this Special Issue.

A masterful clinician also fully comprehends the differing epidemiology, causative agents, and clinical course of these autoimmune bullous dermatoses. Certain dermatoses disproportionately affect select demographic groups, such as the elderly in bullous pemphigoid [10]. Drug-induced vesiculobullous dermatoses are common, and the isolation of the inciting medication from a thorough patient history is important in these cases. Classic causative agents are reviewed, and newer drugs are expounded on, such as the rise of targeted cancer therapeutics such as pembrolizumab and their implicated role in autoimmune bullous dermatoses such as SJS/TEN [11]. Noting the clinical course and timeline of disease is also relevant for diagnosis, and this can be seen in diagnostic criteria, such as the EuroSCAR criteria for AGEP [12]. These important aspects of vesiculobullous dermatoses are elaborated on further in this Special Issue and help add to the clinician’s armamentarium for making diagnoses.

Recognizing key clinical presentations and gathering a pertinent history are vital for a focused patient work-up. This Special Issue summarizes common approaches for diagnostic testing in vesiculobullous dermatoses. The proper utilization of skin biopsies for light microscopy, direct immunofluorescence, and indirect immunofluorescence are reviewed. Modern technologies such as enzyme-linked immunosorbent assays (ELISA) and their increased availability to clinicians has improved serologic testing for dermatoses such as pemphigus, where immunoglobulins against desmogleins can be tracked to monitor disease activity [13,14]. Multiple avenues for diagnosis are explored, such as in paraneoplastic pemphigus where testing for antibodies against plakins is difficult, and alternative options such as indirect immunofluorescence against rat bladder substrate and immunoblotting are utilized [15]. Advanced endoscopic methods for the diagnosis of pemphigus vulgaris are reviewed as well. Importantly, biopsies should serve to help support the clinical diagnosis but cannot be its sole determinant, especially in dermatoses that cannot be distinguished reliably under the microscope such as SJS/TEN, erythema multiforme, and fixed drug eruption. Clinical competence can help reduce the overutilization of medical resources, such as ordering unnecessary labs, and help prevent delays in treatment.

The management of vesiculobullous dermatoses is discussed in-depth within this Special Issue, including new advancements in treating autoimmune bullous disease. Classic standard of care treatments are reviewed, such as dapsone for dermatitis herpetiformis [16]. The advent of corticosteroids has improved mortality rates for many dermatoses, and it remains a crucial aspect of care for diseases such as pemphigoid and pemphigus [17,18]. However, clinicians should plan for a transition to nonsteroidal therapy to avoid side effects from chronic corticosteroid use. Common alternatives include mycophenolate mofetil, methotrexate, azathioprine, intravenous immunoglobulin, and cyclosporine. Rituximab, a monoclonal antibody against CD20+ B-cells, has been approved as a first-line treatment for moderate-to-severe pemphigus foliaceus and pemphigus vulgaris and offers an excellent steroid-sparing alternative for these diseases [19]. Importantly, new research is till advancing our understanding of the pathogenesis of SJS/TEN. Previously hypothesized to mainly involve the Fas/Fas ligand pathway, more recent studies have shown granulysin to be a key mediator in the keratinocyte necrosis seen in SJS/TEN [20]. Expanding our understanding of SJS/TEN is important in finding better treatments targeting its pathological process. Treatments currently revolve around immunosuppressive therapies, including systemic corticosteroids, intravenous immunoglobulin (IVIg), cyclosporine, and TNF-alpha inhibitors [21,22]. Controversy remains regarding which therapies provide a mortality benefit.

The goal of this Special Issue is to simplify the management of autoimmune bullous dermatoses, a category of disease often classified under “complex medical dermatology.” The articles herein offer readers a comprehensive understanding of how vesiculobullous dermatoses can present, how to approach their diagnostic work-up, and offers first-line and alternative treatment options. Mastery of these aspects can simplify a field that is otherwise considered complex and ultimately improve patient care.

## Data Availability

Not applicable.
